# Paclitaxel-loaded ROS-responsive nanoparticles for head and neck cancer therapy

**DOI:** 10.1080/10717544.2023.2189106

**Published:** 2023-03-14

**Authors:** Yaqin Tu, Wei Zhang, Guorun Fan, Chenming Zou, Jie Zhang, Nan Wu, Jiahui Ding, Wen Qing Zou, Hongjun Xiao, Songwei Tan

**Affiliations:** a Department of Otorhinolaryngology, Union Hospital, Tongji Medical College, Huazhong University of Science and Technology, Wuhan, China; b School of Pharmacy, Tongji Medical College, Huazhong University of Science and Technology, Wuhan, China

**Keywords:** ROS, head and neck cancer, paclitaxel, controlled release, targeting

## Abstract

High intracellular reactive oxygen species (ROS) level is characteristic of cancer cells and could act as a target for the efficient targeted drug delivery for cancer treatment. Consequently, biomaterials that react to excessive levels of ROS are essential for biomedical applications. In this study, a novel ROS-responsive polymer based on D-α-Tocopheryl polyethylene glycol 1000 succinate (TPGS) and poly (β-thioester) (TPGS-PBTE) was synthesized for targeted delivery of the first-line antineoplastic drug, paclitaxel (PTX). The resultant TPGS-PBTE NPs showed good ROS-responsive capability in size change and drug release. Compared to PTX, PTX-loaded nanoparticles (PTX@TPGS-PBTE NPs) showed enhanced cytotoxicity and higher level of apoptosis toward squamous cell carcinoma (SCC-7) cells. Tumor-targeted delivery of the NPs was also observed, especially after being modified with a tumor-targeting peptide, cRGD. Enhanced tumor growth inhibition was also observed in head and neck cancer SCC-7 murine models. In summary, PTX@TPGS-PBTE NPs can achieve good therapeutic effects of PTX against head and neck cancer both in vitro and in vivo, especially when modified by cRGD for active targeting, which enriched the application of ROS responsive system utilized in the delivery of anticancer drugs.

## Introduction

Recently, nanoparticle (NP)-based delivery systems for cancer therapy have attracted extensive research attention due to their improved therapeutic efficacy and reduced side effects (Iyer et al., 2006; Bertrand et al., 2014; Li et al., 2021; 2022a, 2022b). However, the insufficient release of nanomedicines in cells limits their clinical application (Fang et al., 2011; Sun et al., 2014). It is widely accepted that highly proliferative cancer cells produce elevated amounts of reactive oxygen species (ROS) compared to healthy cells. Cancer sites can be distinguished from their surrounding tissues due to the elevated level of intracellular ROS. Based on this, controlled drug release at target sites triggered by intracellular stimuli, such as ROS, is likely to be required to achieve sufficient drug levels (Zhao et al., 2013).

Given the pathological ROS levels in cancer cells, researchers have been increasingly interested in the application of ROS-responsive drug delivery systems for tumor therapy (Saravanakumar et al., 2017; Li et al., 2020; Liang et al., 2021; Mollazadeh et al., 2021). Various ROS-responsive functional groups, such as prodrugs or carriers that have been utilized in the application of smart drug delivery systems, including structures that contain thioketal, thioether, monoselenide/diselenide, telluride, arylboronic ester, aminoacrylate, oligoproline, and peroxalate ester, have been employed in the development of ROS-responsive drug delivery systems (Saravanakumar et al., 2017; Tao & He, 2018). Among them, thioether is a widely used ROS-responsive functional group with broad ROS species responsivity. Its oxidation products are sulfoxide or sulfone; these are much more hydrophobic than the original thioether, inducing a hydrophobic-hydrophobic phase transition of thioether-containing polymers. Moreover, the formation of sulfone promotes the hydrolysis of the proximal ester bond (Luo et al., 2016; Tan et al., 2022). Thus, a ROS-accelerated drug release will be achieved with thioether-based polymers or prodrugs.

**Scheme 1.**
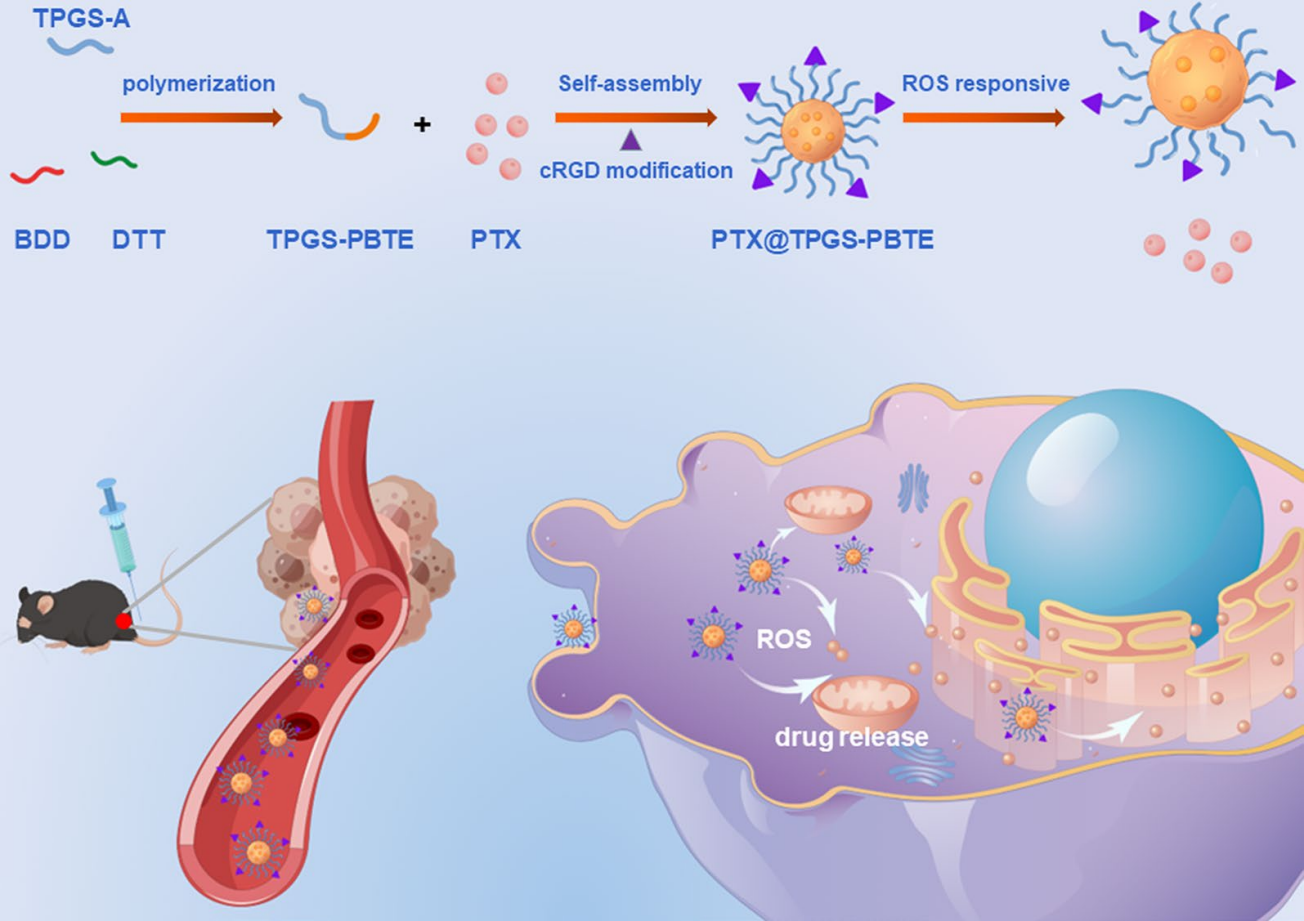
Preparation of PTX@TPGS-PBTE NPs and schematic illustration of tumor treatment mechanism.

D-α-Tocopheryl polyethylene glycol 1000 succinate (TPGS) is a water-soluble derivative of natural vitamin E and polyethylene glycol (PEG) 1000. It has been approved by the Food and Drug Administration (FDA) (Zhang et al., 2012; Guo et al., 2013; Liang et al., 2018; Yang et al., 2018) for use as an adjuvant pharmaceutical. TPGS consists of α-TOS and polyethylene glycol (PEG) and is usually utilized to achieve a long circulation time of nanoparticles (NPs). Research has also shown that TPGS is cytotoxic to some tumor cells due to intracellular ROS elevation and mitochondrial-associated apoptosis (Youk et al., 2005; Tan et al., 2017; Guan et al., 2020), which makes TPGS a powerful candidate for anti-tumor drug delivery systems and a potential inductive agent to accelerate the ROS-responsive drug release in tumor cells. In this work, TPGS, dithiothreitol (DTT), and 1, 4-butanediol diacrylate (BDD) were used to synthesize a biocompatible and ROS-responsive thioether-containing copolymer, termed poly (β-thioester) (TPGS-PBTE), via fast Michael addition polymerization (Scheme 1). TPGS acts as the hydrophilic segment and thus improves the system stability of the NPs. PBTE was used to encapsulate an anti-tumor drug in order to achieve a ROS-triggered release. Paclitaxel (PTX), a first-line antineoplastic drug with excellent efficacy against cancers of the head and neck and breast, was chosen as the model drug in this study (Abu Samaan et al., 2019; Cui et al., 2020; Haider et al., 2020). Then, PTX was loaded to form PTX@TPGS-PBTE NPs. After being uptaken by squamous cell carcinoma SCC-7 cells, TPGS induced the production of excess intracellular ROS to accelerate the release of PTX due to the ROS responsivity of the thioester bond (Lippert et al., 2011). In addition, the NPs can be decorated with specific ligands, cRGD, for enhanced cancer cell targeting and decreased off-target effects (Toyokuni et al., 1995; Song et al., 2014). Therefore, the developed PTX@TPGS-PBTE NPs offered passive targeting, self-promoted drug release and reduced side effects, which can enhance the chemotherapy efficacy of PTX.

## 2. Materials and methods

### 2.1. Materials

PTX and DTT were purchased from Aladdin (Shanghai, China). BDD was obtained from TCI (Shanghai, China). RPMI-1640 medium, fetal bovine serum (FBS), penicillin-streptomycin, and trypsin EDTA were obtained from Gibco Life Technologies (AG, USA). A Cell counting Kit-8 and Calcein-AM/PI double staining kit were purchased from Beyotime Biotechnology (China). A BD Annexin V-FITC apoptosis detection kit was purchased from BD Biosciences (New York). The solvents used were all analytical grades and obtained from Sinopharm (China). All animal protocols were performed at the Huazhong University of Science and Technology. They were maintained at the Laboratory Animal Center of the Huazhong University of Science and Technology with standard access to water and food. Animal experiments were approved by the Experimental Animal Ethics Committee of Tongji Medical College of Huazhong University of Science and Technology (IACUC Number: 3128).

### 2.2. Synthesis and characterization of TPGS-PBTE

First, TPGS was esterified with acryloyl chloride to form TPGS acrylate. Then, 158 mg of TPGS acrylate (0.1 mM) in 3 mL N, N-dimethylformamide (DMF) was co-dissolved with trace hexylamine in 396 mg BDD (2 mM) and 317 mg DTT (2.1 mM). After reacting at room temperature overnight, dialysis of the solution was performed twice in a water/DMF mixture (50:50, v:v) and three times in water using a dialysis tube (MWCO 3500). The final TPGS-PBTE product was lyophilized and characterized using ^1^HNMR (Bruker AVANCE III 400 MHz NMR spectrometer, solvent: CDCl_3_) and FT-IR (PerkinElmer, Spectrum Two).

### 2.3. Preparation of PTX@TPGS-PBTE NPs

PTX@TPGS-PBTE NPs were synthesized by the nanoprecipitation – dialysis method. Briefly, 9 mg TPGS-PBTE, 1 mg DSPE-PEG, and 2 mg PTX were co-dissolved in DMF (1.5 mL) and then added in a dropwise manner into water under stirring. The NPs solution was centrifugated at 500 rpm for 3 min to remove unloaded PEX and aggregations. To prepare the cRGD-modified NPs, DSPE-PEG-Mal was used instead of DSPE-PEG and then the NPs solution was incubated with cRGD peptide (equal molar amount of DSPE-PEG-Mal) at room temperature for 4 h. The free cRGD was centrifuged in a Millipore microdialyzer (MWCO 10 kDa) at 3000 rpm for 40 min, as described in our previous work (Bao et al., 2016). Then, TPGS-PBTE labeled with DiR iodide (DiR@TPGS-PBTE) and Coumarin-6 (C6@TPGS-PBTE), respectively, was prepared using similar procedures to those used for PTX@TPGS-PBTE.

### 2.4. Characterization of PTX@TPGS-PBTE NPs

Transmission electron microscopy (TEM) and dynamic light scattering (DLS) techniques were applied to the PTX@TPGS-PBTE to determine the size and morphology of the NPs, respectively. Moreover, PTX@TPGS-PBTE was diluted in FBS or Phosphate-Buffered Saline (PBS) and maintained at 37 °C for stability testing. The critical aggregation concentration (CAC) of the NPs was determined using pyrene (Bao et al., 2014). The amount of PTX encapsulated in the NP was measured by high-performance liquid chromatography (HPLC) (Hitachi 2000, Japan) equipped with a UV detector and a reverse phase Inertsil ODS-3 C18 column (150 × 4.6 mm, pore size 5 μm, Agilent, USA). To measure the loading ability of PTX in the NPs, freshly prepared PTX@TPGS-PBTE NPs solution was lyophilized and redissolved in the methyl alcohol. The solution was then filtered by 0.45 μm filter for HPLC analysis with the mobile phase of acetonitrile/water solution (50/50, v/v), flow rate of 1 mL/min (isocratic elution mode), and detector UV wavelength of 227 nm. The encapsulation efficiency (EE) and drug loading (DL) of PTX were calculated as followed:

(1) $$\mathrm{EE}(\%)=\frac{\text{amount of PTX in NPs}}{\text{feeding amount of PTX}}$$

(2) $$\mathrm{DL}(\%)=\frac{\text{amount of PTX in NPs}}{\text{amount of the NPs}}$$

To explore the redox-responsive properties of PTX@TPGS-PBTE NPs, DLS was used to measure the disassembly of PTX@TPGS-PBTE under stimulation with different concentrations of H_2_O_2_. The in vitro release of PTX from PTX@TPGS-PBTE NPs was detected in pH 7.4 PBS with or without 10 mM H_2_O_2_ as the release media. Briefly, 5 mL of freshly prepared PTX@TPGS-PBTE NPs solution was added into a dialysis tube (MWCO: 3500) and dialyzed against 50 mL of release media at 37 °C with constant shaking. At the predetermined time intervals, 1 mL of the solution was removed and replaced with an equal volume of fresh-release media. The released PTX amount was measured by HPLC as described above.

### 2.5. Cell culture

SCC-7 cells were grown in RPMI-1640 medium containing 10% FBS, 100 U/mL penicillin G sodium, and 100 μg/mL streptomycin sulfate. The cells were incubated in a humidified incubator at 37 °C with 5% CO_2_.

### 2.6. Intracellular uptake

Fluorescence microscopy was performed using Coumarin-6 as a fluorescent probe in order to visualize cellular uptake. SCC-7 cells were seeded in a 24-well plate at a density of 1.0 × 10^4^ cells per well. After 24 h attachment, the cells were incubated at 37 °C for 0, 0.5, 1, 2, and 4 h in medium containing Coumarin-6@TPGS-PBTE. The cells were then rinsed with PBS, fixed in 4% paraformaldehyde, and stained with DAPI. The mean fluorescence intensity was analyzed with ImageJ software.

### 2.7. In vitro cytotoxicity and apoptosis

The SCC-7 cell line was used to assess the cytotoxicity of Taxol and PTX@TPGS-PBTE NPs. Briefly, SCC-7 cells were seeded into separate 96-well plates and were cultured for 24 h. Subsequently, the medium was replaced with fresh medium containing PTX (0.1, 0.5, 1.0, 5.0, and 10.0 g/mL), with an exposure duration of 24 or 48 h. SPSS software (19.0) was used to calculate the IC_50_ value (concentration inhibiting cell growth by 50%). Cell apoptosis was assessed by Calcein-AM and propodium iodide (PI) staining. Calcein-AM and PI were used to stain and detect live and dead cells in each group for 30 min by fluorescence microscope. Apoptosis was analyzed using flow cytometry. The cells were plated overnight in 5% CO_2_ at 37 °C and seeded at 10^5^ cells per well. Then, the adherent cells were treated with Taxol and PTX@TPGS-PBTE. After redispersing the cells, the live and dead cells were labeled with Annexin V (5 μL) and PI (5 μL), respectively, for 15 min. Finally, cell apoptosis was detected with a flow cytometer.

### 2.8. Biodistribution study

For the biodistribution analysis, SCC-7 tumor-bearing mice were administered free DIR, DIR@TPGS-PBTE, and RGD-DIR@TPGS-PBTE NPs intravenously (i.v.). The mice were divided randomly into three groups after the tumor volume reached 100 mm^3^. Each group was treated (i.v.) with free DIR, DIR@TPGS-PBTE, and RGD-DIR@TPGS-PBTE, respectively. Then, three mice in each group were sacrificed at intervals of 2, 4, 8, 12, 24, 48, 72, and 120 h after injection, and the tumors and major organs were collected. The fluorescence intensity of the tumors was assessed via autofluorescence using an in vivo imaging system (IVIS, Caliper, USA).

### 2.9. Efficacy and safety evaluations in vivo

In vivo therapeutic efficacy was evaluated in SCC-7 tumor-bearing mice. Upon tumor growth to 50–100 mm^3^, the mice were randomly divided into four groups (six mice each): (1) saline, (2) Taxol, (3) PTX@TPGS-PBTE, and (4) RGD-PTX@TPGS-PBTE. The tumor size and body weight measurements were taken every two days. Sixteen days after commencing treatment, the mice were euthanized. Tumor inhibition rate (TIR) was obtained from the tumor weight: TIR (%) = [1 − tumor weight of experimental group/tumor weight of control group (N.S. treated)] × 100%. The tumors and major organs were excised for hematoxylin and eosin (H&E) staining and TUNEL staining for apoptotic tumor cells. The blood vessels were stained with anti-CD31 antibodies. The nuclei were stained with DAPI, and the sections were observed by fluorescent microscopy. The serum from each mouse was collected for biochemistry testing. Albumin (ALB), globulin (GLO), the albumin-globulin ratio, and urea (URE) levels were used to assess liver and kidney functions.

### 2.10. Statistical analyses

The results are expressed as the mean ± standard deviation (SD). Differences between two groups were evaluated with Student’s *t*-tests while multi-group differences were evaluated with two-way ANOVA. The significance threshold was set at *P* < .05.

## 3. Results and discussion

### 3.1. Synthesis and characterization of TPGS-PBTE

**Figure 1.**
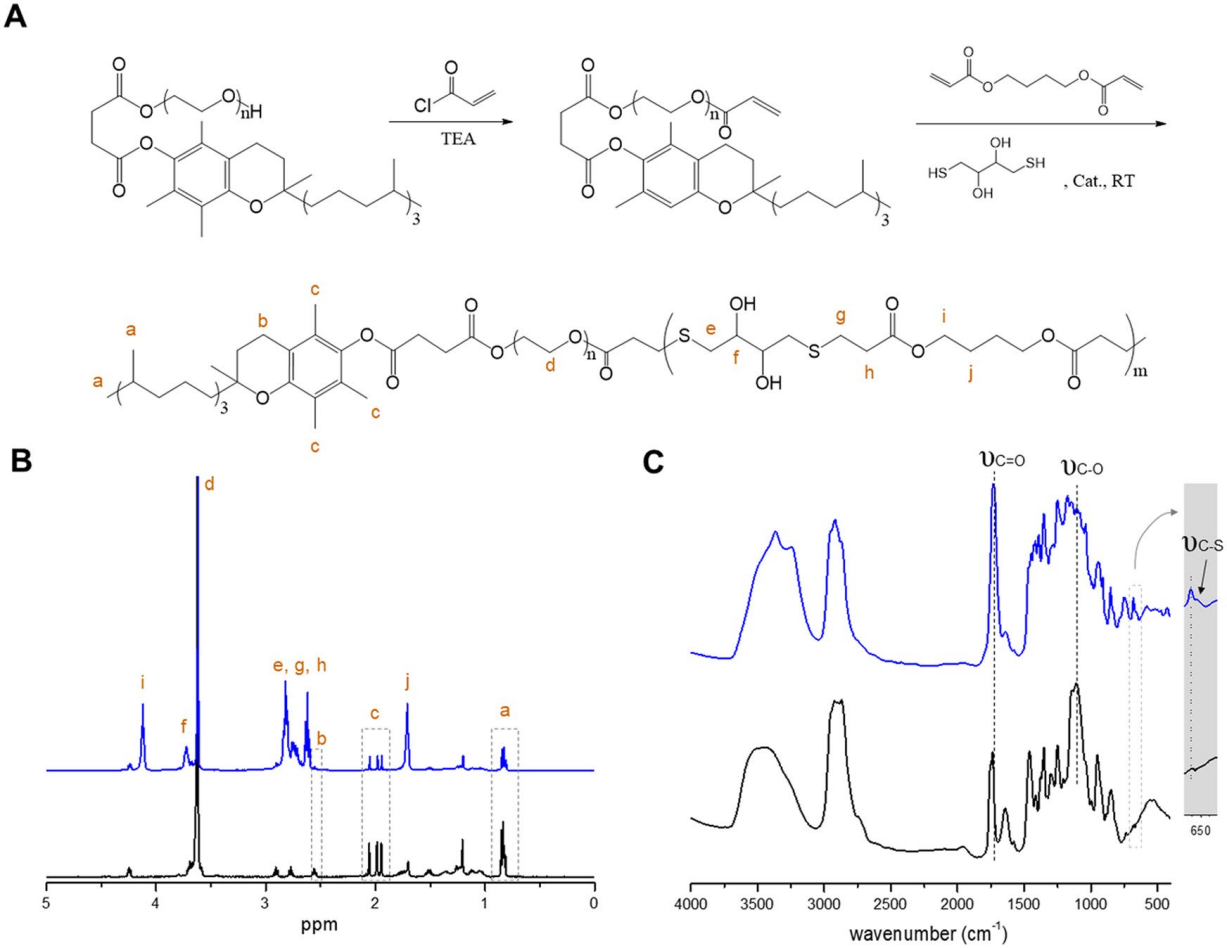
Synthesis and characterization of TPGS-PBTE. (A) Synthetic scheme of TPGS-PBTE. (B) ^1^H NMR and (C) FTIR spectra of TPGS (black) and TPGS-PBTE (blue).

The ROS-responsive TPGS-PBTE was first prepared by acrylic esterification of TPGS to form the macro-monomer, TPGS acrylic ester, and then polymerized via a Michael addition reaction of BDD, DTT, and TPGS acrylic ester with acrylate-thiol at a molar ratio of 1:1.05 (Figure 1A). The products were verified by ^1^H-NMR and FTIR. As shown in Figure 1B, typical spectral peaks of TPGS (0.86, 1.90–2.10, and 2.56 ppm), BDD (1.71 and 4.12 ppm), and DTT (3.72 ppm) were observed, indicating the successful synthesis of TPGS-PBTE. The structure of TPGS-PBTE was further validated by FT-IR (Figure 1C). The results revealed the typical stretching vibration peak of the C=O bond (υ_C=O_) at 1740 cm^−1^ in BDD, the stretching vibration peak of the C─O bond (υ_C–O_) at 1105 cm^−1^ in TPGS and the stretching vibration peak of the C─S bond (υ_C–S_) at 660 cm^−1^ in DTT (Sheppard, 1950). Moreover, an enhanced broad peak (3200–3700 cm^−1^) from the –OH group was identified. These results confirm the existence of TPGS, BDD, and DTT moieties. These results further illustrated the formation of TPGS-PBTE.

### 3.2. Characterization of PTX@TPGS-PBTE NPs

**Figure 2.**
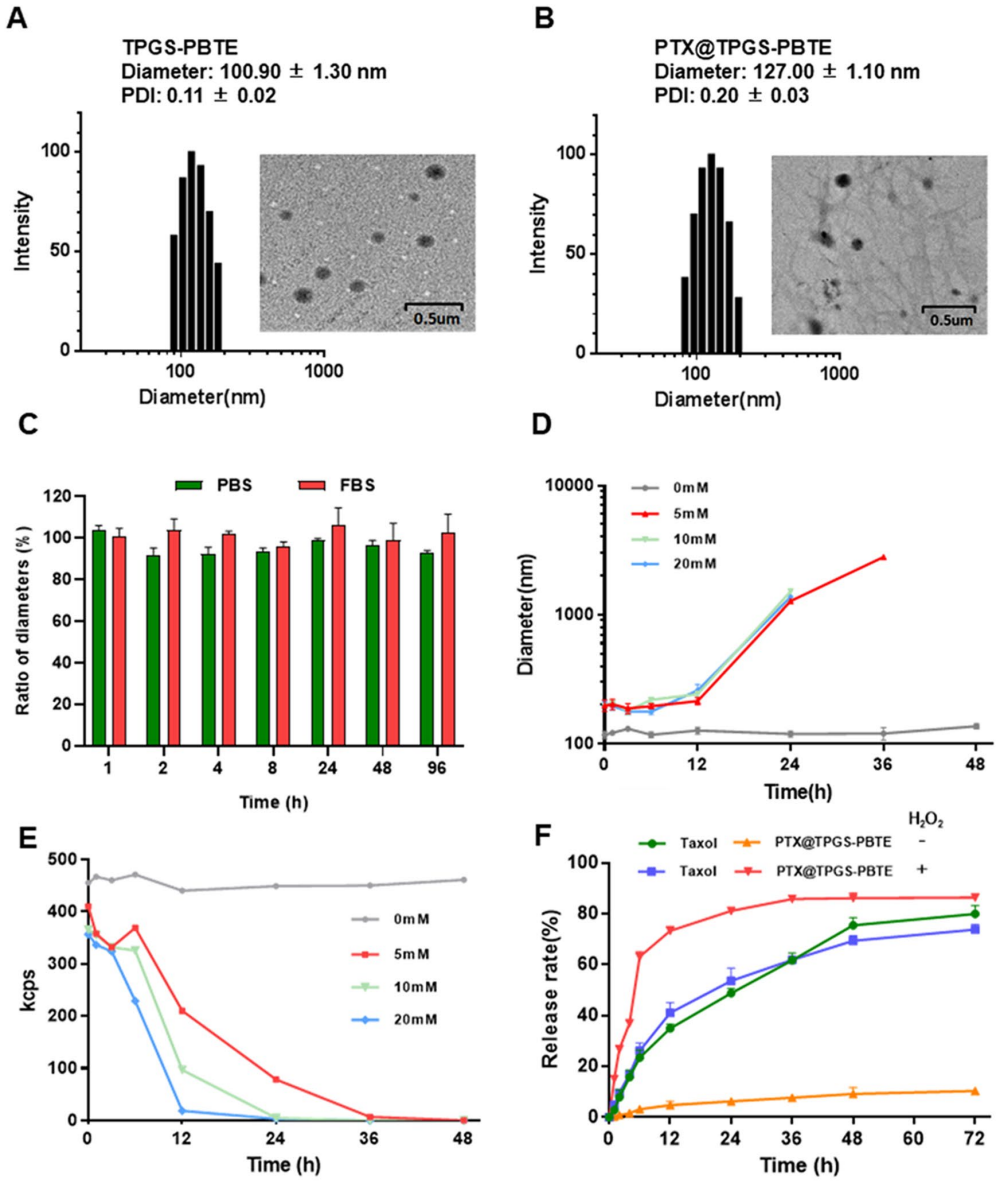
Characterization of PTX@TPGS-PBTE NPs. DLS results and TEM image of (A) TPGS-PBTE and (B) PTX@TPGS-PBTE. (C) Stability and (D, E) ROS-responsibility of PTX@TPGS-PBTE. (F) Drug release of PTX@TPGS-PBTE *in vitro.*

The PTX-loaded TPGS-PBTE NPs were synthesized by the nanoprecipitation – dialysis method with an EE of 75.53% and DL of 11.54%. As shown in Figure 2A, B, the particle sizes of TPGS-PBTE and PTX@TPGS-PBTE NPs measured by DLS were 100.90 ± 1.30 nm (PDI = 0.11 ± 0.02) and 127.00 ± 1.10 nm (PDI = 0.20 ± 0.03), respectively. The TPGS-PBTE and PTX@TPGS-PBTE NPs appeared to be uniform spherical shapes with diameters of 100.00 nm and 115.40 nm, respectively, according to the TEM images. In comparison to the TEM results, the particle size as observed by DLS was a little larger. This is probably due to the presence of a solvation layer on the particle surface and the solvation layer may impact the observed particle size (Wu et al., 2022). The particle size of PTX@TPGS-PBTE remained stable during incubation in PBS and FBS for 96 h at 37 °C, indicating good stability of PTX@TPGS-PBTE under physiological conditions (Figure 2C).

Then, the ROS-responsivity of TPGS-PBTE NPs was evaluated. The time-dependent particle size changes in PTX@TPGS-PBTE NPs under different H_2_O_2_ contents were monitored to verify the responsivity. The size of the NPs increased slightly for the first 6 h under different concentrations of H_2_O_2_. Then, the particle size of the NPs increased gradually and finally degraded completely after 24 h (10 and 20 mM H_2_O_2_) or 48 h (5 mM H_2_O_2_) incubation (Figure 2D). The current counting rate of TPGS-PBTE NPs also exhibited a decreasing tendency (Figure 2E), reflecting the gradual hydration of the NPs (Tan et al., 2011). This is attributed to the fact that the thioether bond of TPGS-PBTE was oxidized to sulfoxide and thus hydrophobic PBTE segment become hydrophilic when H_2_O_2_ was introduced. So, the NPs gradually swelled with the increase in particle size. The NPs finally dissolved in water as the combined effect of increased hydrophilicity and hydrolysis of PBTE. Thus, it can be concluded that TPGS-PBTE exhibited good ROS responsivity, starting at a relatively low H_2_O_2_ concentration of 5 mM.

To investigate the in vivo ROS-responsive drug release behavior of PTX@TPGS-PBTE, H_2_O_2_ (10 mM) was used as a trigger. Taxol is a clinical formulation of the Cremophor EL (CrEL) compound. It is commonly used to evaluate new PTX formulations (Chiang & Yang, 2021). In Figure 2F, it can be seen that PTX released from Taxol exhibited similar release profiles in 12 h (34.95% and 41.04%) and 72 h (79.94% and 73.76%) with or without H_2_O_2_, indicating a lack of ROS-responsibility of Taxol. For PTX@TPGS-PBTE NPs, the cumulative amount of PTX reached as high as 73.33% in 12 h in the presence of 10 mM H_2_O_2_, while only 4.79% of PTX was released in 12 h and this slowly increased over 72 h under nonoxidative conditions. This demonstrates that the NPs released PTX in an H_2_O_2_-dependent manner. Thus, it can be concluded that PTX@TPGS-PBTE showed stability in a normal physiological environment, but the drug release rate was accelerated in the presence of H_2_O_2_, which reduces the risk of possible side effects. This finding suggests that PTX@TPGS-PBTE NPs could be responsive to the tumor microenvironment (oxidative conditions), which might trigger particle size alterations and efficient drug release.

### 3.3. Intracellular uptake and in vitro cytotoxicity

**Figure 3.**
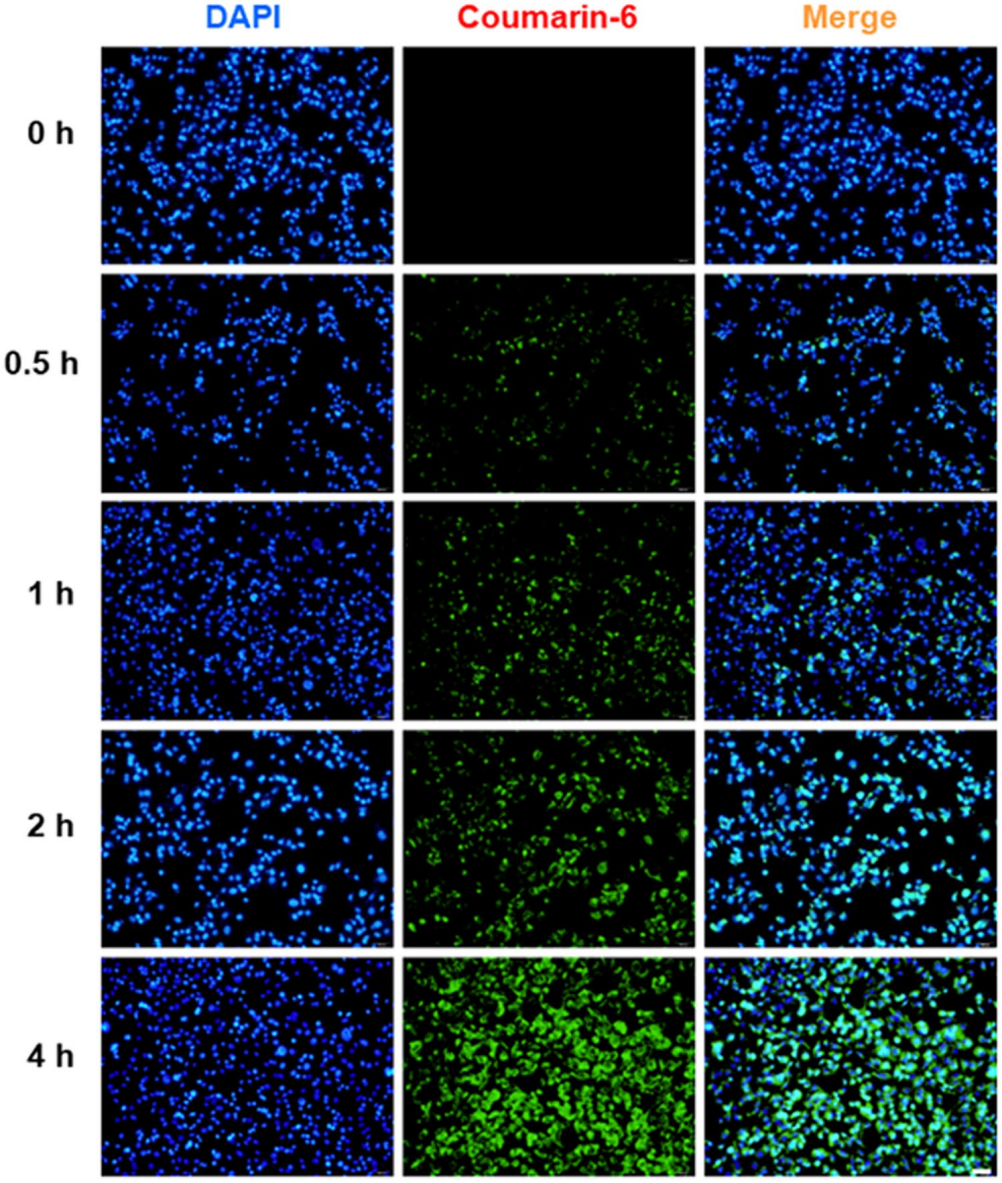
Representative fluorescence images of in vitro cellular uptake of Coumarin-6 labeled TPGS-PBTE NPs in SCC-7 cells at different time intervals.

**Figure 4.**
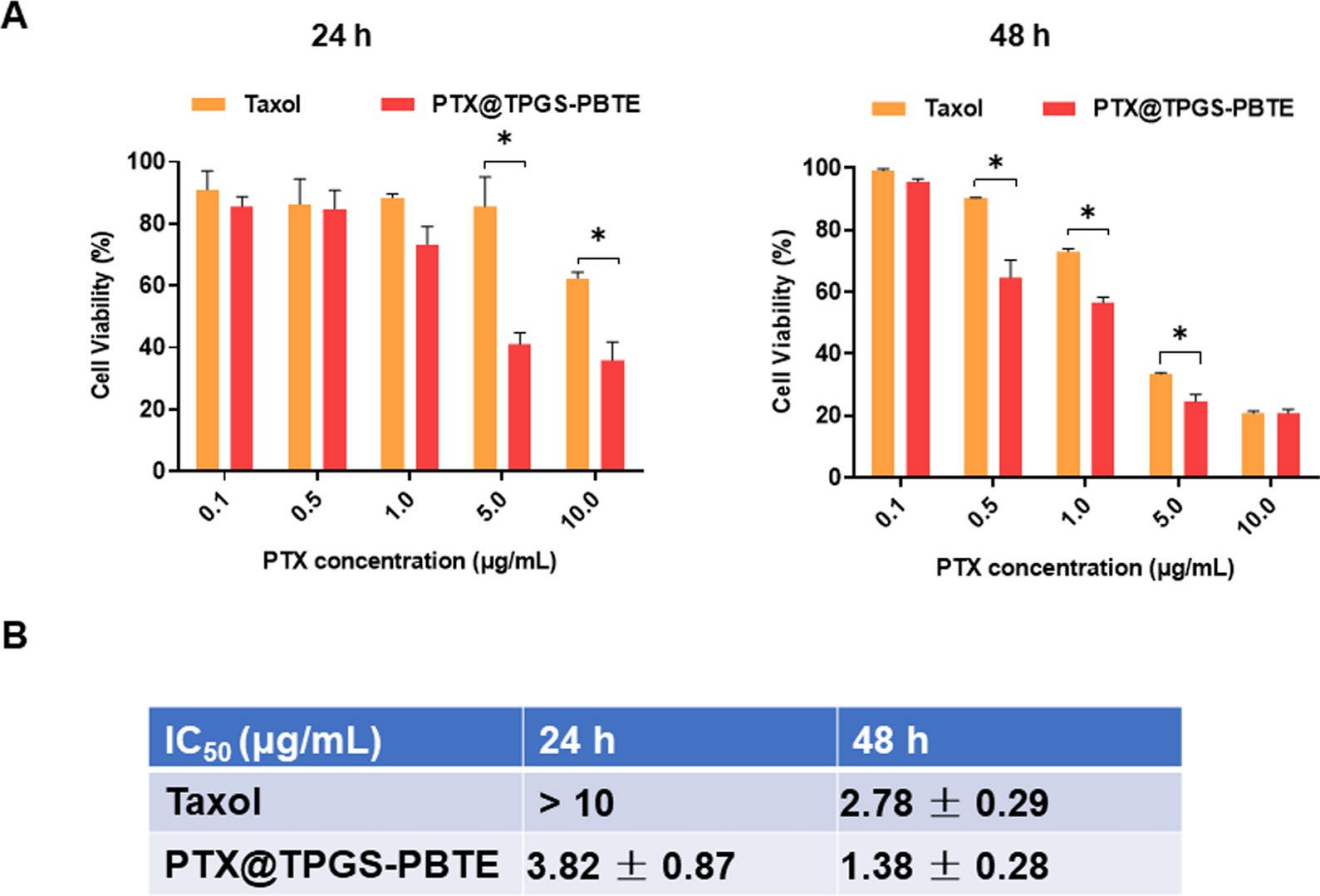
*In vitro* cytotoxicity investigation. (A) In vitro cytotoxicity assays of Taxol and PTX@TPGS-PBTE against SCC-7 cells after treatment for 24 and 48h. *, *p* < .05. (B) The IC_50_ value of Taxol and PTX@TPGS-PBTE NPs against SCC-7 cells.

To confirm the cellular uptake characteristics, Coumarin-6 was used as a probe. SCC-7 cells were incubated with the NPs for various durations (0, 0.5, 1, 2, and 4 h; Figures 3, S1). The results revealed that Coumarin-6 fluorescence was mainly distributed throughout the cytoplasm in SCC-7 cells. Further, the green fluorescence (Coumarin-6) increased over time, suggesting that the NPs loaded with Coumarin-6 were effectively taken up by the cells within 4 h. SCC-7 cells were incubated with Taxol and PTX@TPGS-PBTE at gradient concentrations for 24 h and 48 h. As shown in Figure 4A, significant cytotoxicity of SCC-7 cells was observed with both Taxol and PTX@TPGS-PBTE, depending on the drug concentration and incubation time. The cell viability of Taxol was still nearly 62.45% at 24 h, even with the highest PTX concentration. This indicates that Taxol had a limited cytotoxic effect in the first 24 h. The cytotoxicity of PTX@TPGS-PBTE was lower than that of Taxol, implying that PTX@TPGS-PBTE could somewhat enhance the cytotoxicity of PTX. The IC_50_ was further quantified to evaluate the anticancer effects of Taxol and PTX@TPGS-PBTE (Figure 4B). The IC_50_ values of PTX@TPGS-PBTE against SCC-7 cells were observed to be 3.82 ± 0.87 and 1.38 ± 0.28 μg/mL after 24 and 48 h of treatment, respectively, which were significantly lower than those of Taxol. These findings further demonstrated that the PTX@TPGS-PBTE NPs exhibited a stronger ability to destroy SCC-7 cells than Taxol due to the superior ROS-responsive drug release ability of PTX@TPGS-PBTE. We further studied the changes in intracellular ROS, as shown in Figure S2. TPGS had an obvious intracellular ROS response, but in PTX@TPGS-PBTE group, the ROS increase was greatly inhibited. This indicated that PBTE may consume the excess ROS triggered by TPGS by the oxidation reaction of the thioether bond, thus resulting in self-promoted ROS-responsive drug release in tumor cells.

**Figure 5.**
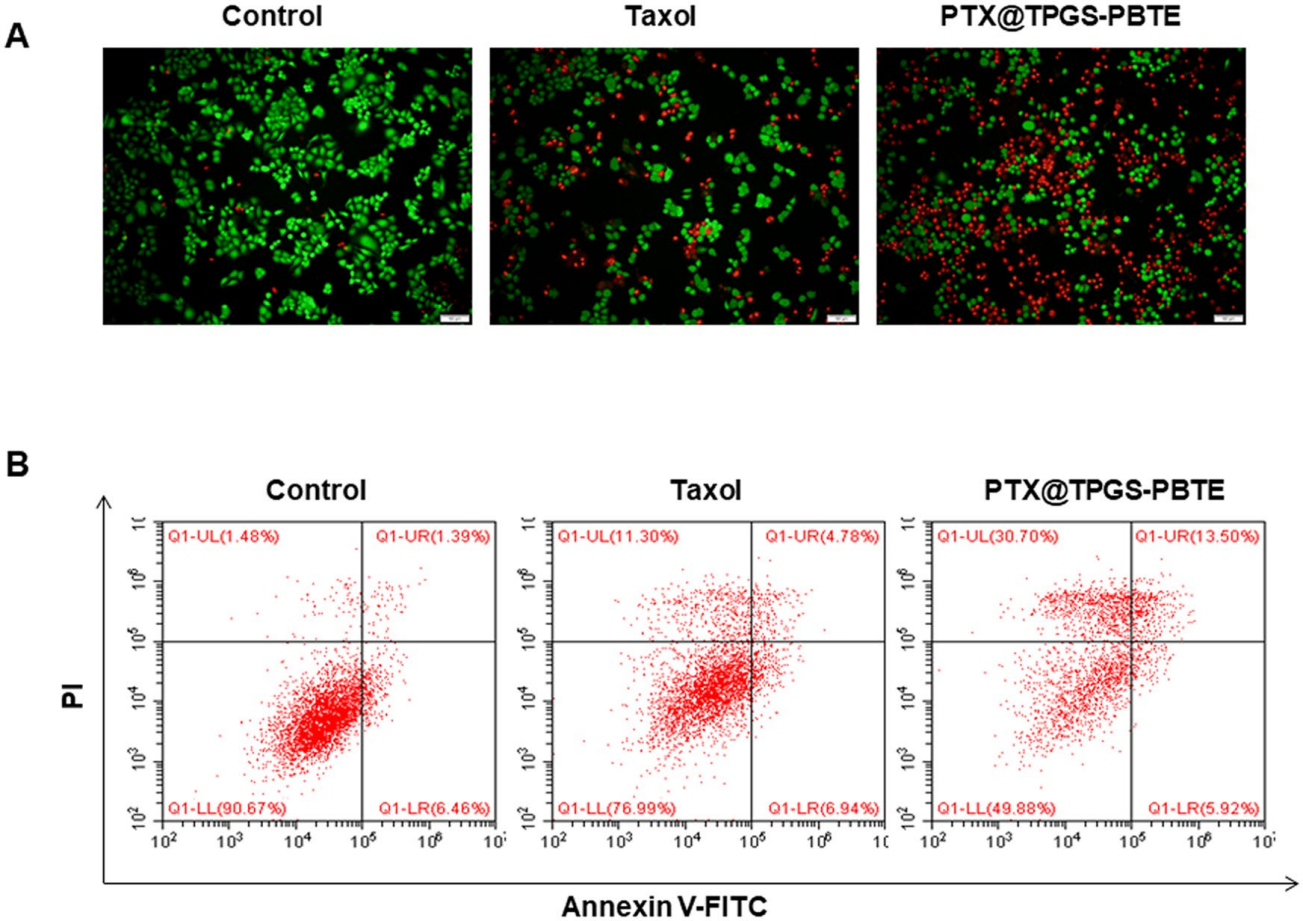
Apoptosis investigation. (A) The apoptosis of SCC-7 cells after various treatments was detected by Calcein AM/PI staining (Green: live cells, Red: dead cells. Scale bar: 100 μm.); (B) Annexin V-FITC/PI double staining by flow cytometry.

The cytotoxicity of PTX@TPGS-PBTE and Taxol was further determined by fluorescence staining of live and dead SCC-7 cells. Calcein-AM and PI were used to visualize the live (green) and dead cells (red), respectively (Figure 5A). Consistent with the CCK-8 results, incubation with PTX@TPGS-PBTE would induce the death of the vast majority of cells. In contrast, the Taxol treatment group showed less detectable damage. This provided strong evidence of the excellent anti-tumor efficiency of PTX@TPGS-PBTE on SCC-7 cells. In addition, the flow cytometry analysis showed that the percentages of early apoptotic cells (Q1-LR) were similar between Taxol and PTX@TPGS-PBTE treatments, while the percentages of late apoptotic cells (Q1-UR) and dead cells (Q1-UL) in PTX@TPGS-PBTE treatment group (13.50% and 30.70%) were much higher than those of Taxol (4.78% and 11.30%) (Figure 5B). Together, these results indicated that PTX@TPGS-PBTE was more effective than Taxol at inhibiting SCC-7 cell growth.

### 3.4. Biodistribution of PTX@TPGS-PBTE NPs

**Figure 6.**
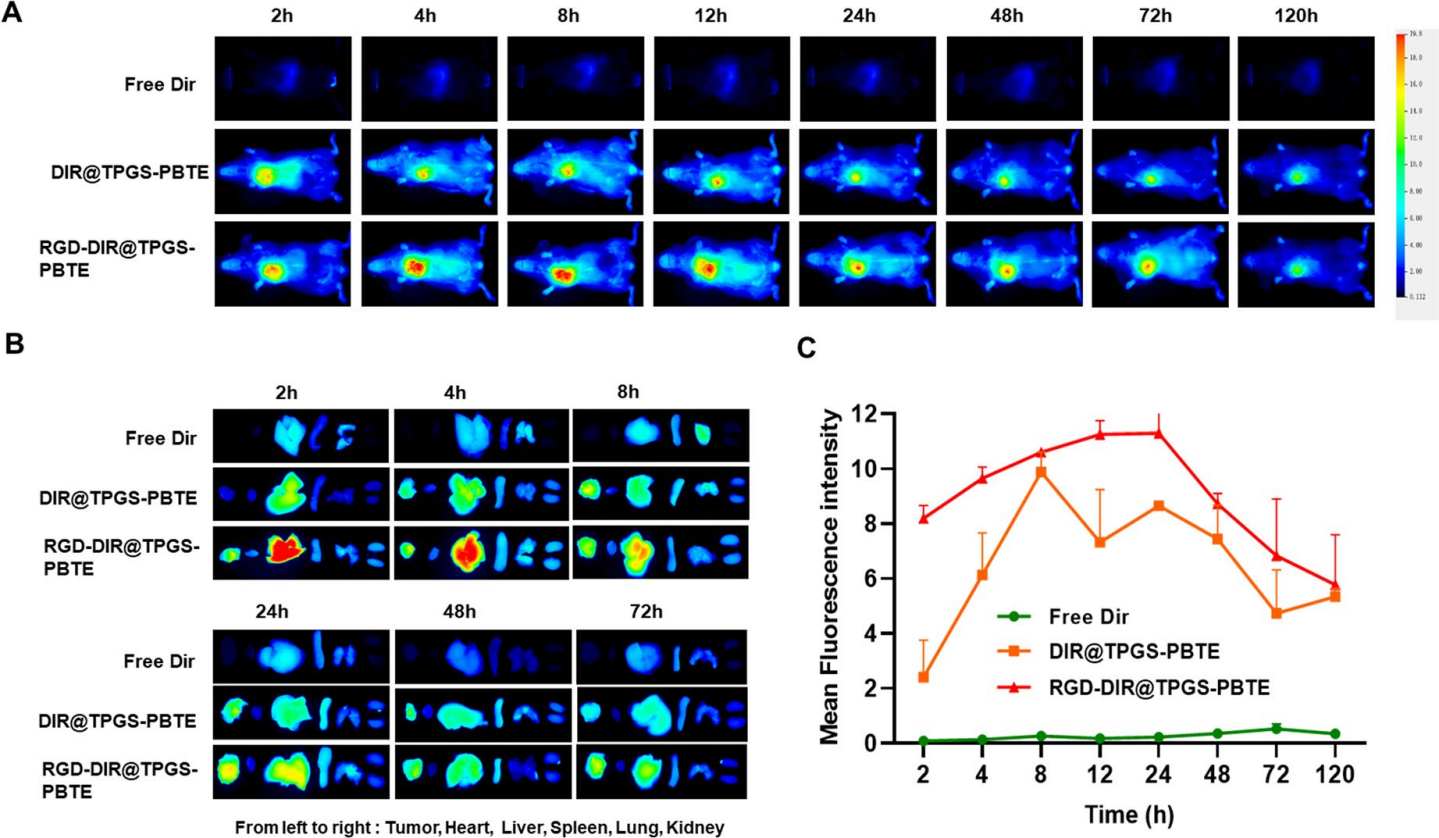
*In vivo* pharmacokinetic and biodistribution study. (A) Fluorescence images of SCC-7 tumor-bearing mice after i.v. injection of free DIR, DIR@TPGS-PBTE, and RGD-DIR@TPGS-PBTE at different time intervals; (B) Fluorescence imaging of major organs and tumors at different time intervals. (C) Fluorescence intensity-Time profiles of free DIR, DIR@TPGS-PBTE, and RGD-DIR@TPGS-PBTE in SCC-7 tumor.

The biodistribution of PTX@TPGS-PBTE NPs was examined in SCC-7-bearing mice. The short peptide, cRGD, consists of arginine-glycine-aspartate (Arg-Gly-Asp) peptide sequences and is often used as a homing peptide to enhance tumor-targeting and tumor-penetrating capabilities (Min et al., 2020; Zhang et al., 2020; Cheng et al., 2021). Thus, it can be used to endow NPs with tumor tissue targeting capability and thus the targeting NPs (RGD-DIR@TPGS-PBTE) was developed. As shown in Figure 6(A), DIR@TPGS-PBTE NPs and RGD-DIR@TPGS-PBTE NPs displayed significantly enhanced tumor accumulation compared with free DIR. The fluorescence signals of DIR@TPGS-PBTE NPs and RGD-DIR@TPGS-PBTE NPs increased gradually in tumor areas, reaching a maximum of 8–24 h after injection, and then gradually decreased. In contrast, the free DIR group exhibited rapidly reduced fluorescence signals without showing specific aggregation in tumor regions, exhibiting a fast clearance rate and a low tumor-targeting ability. According to the above results, DIR@TPGS-PBTE NPs and RGD-DIR@TPGS-PBTE NPs exhibited prominent tumor retention, especially the cRGD-modified group. This indicated that cRGD peptides enhanced the enrichment of NPs in tumor tissue by targeted modification. This accumulation may be a result of their superior tumor-targeting ability and enhanced permeability and retention (EPR) effect (Sun et al., 2017; Gong et al., 2021). In order to validate this tumor accumulation, tumor-bearing mice were euthanized at various time points and their major organs were collected for ex vivo imaging. Consistent with the above results, DIR@TPGS-PBTE NPs and RGD-DIR@TPGS-PBTE NPs exhibited greater accumulation in the tumor region than free DIR (Figure 6B). The area under the curve (AUC_0-t_) values were 22.34 ± 5.58, 530.48 ± 64.55, and 676.27 ± 55.98 mg/L•h for free DIR, DIR@TPGS-PBTE NPs, and RGD-DIR@TPGS-PBTE NPs, respectively, and the *t*_1/2_ values were 50.56 ± 25.88, 62.01 ± 23.50, and 124.77 ± 56.74 h, respectively (Figure 6C). These results indicated that TPGS-PBTE can achieve tumor accumulation via passive targeting, and this can be further enhanced by active targeting (cRGD) modification.

### 3.5. In vivo anti-tumor efficacy and biosafety evaluation

**Figure 7.**
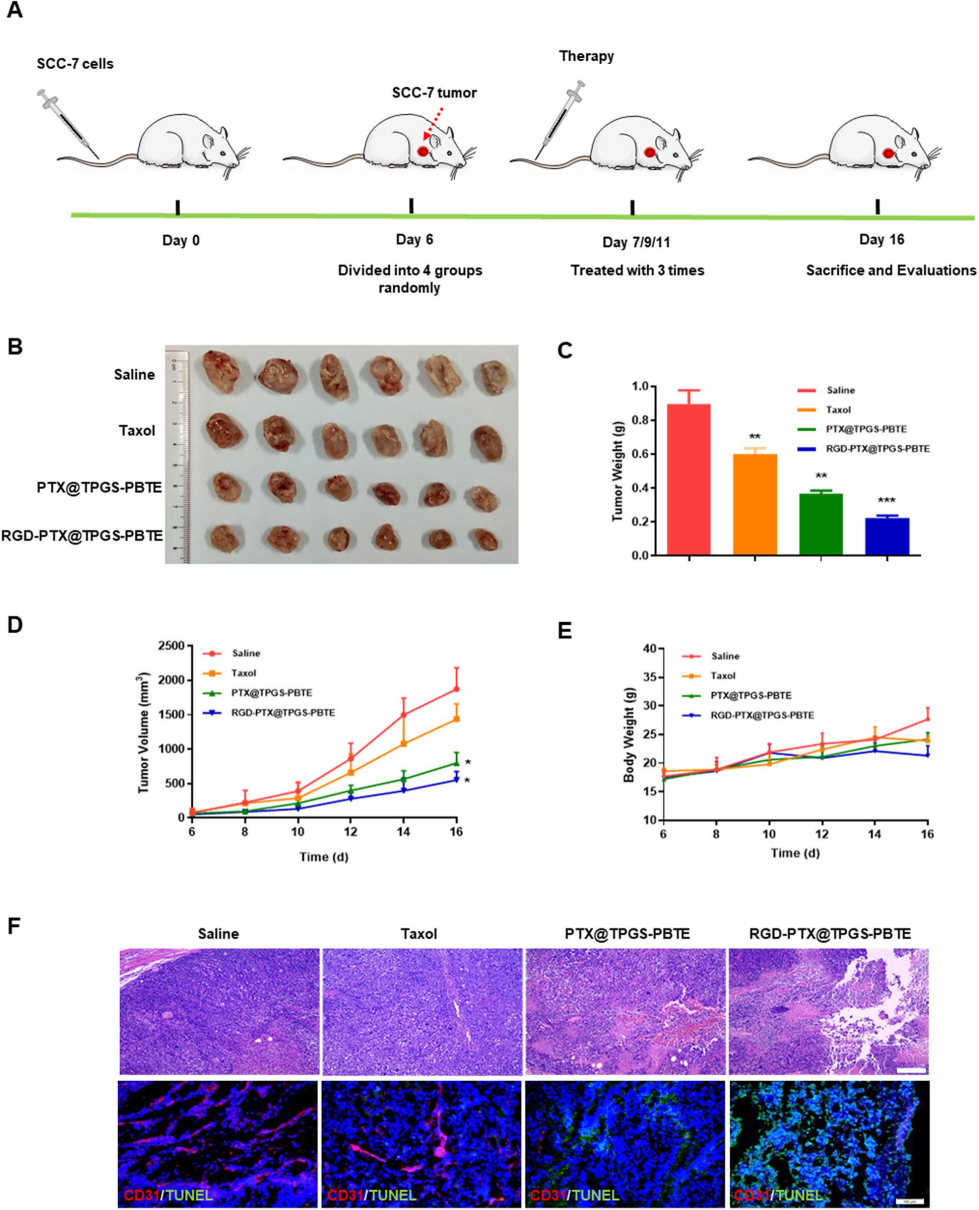
In vivo therapeutic effects in tumor-bearing mice. (A) Schematic illustration of in vivo antitumour treatment; (B) Ex vivo images of tumor tissues; (C) Tumor weight, (D) tumor volume, and (E) body weight of SCC-7 tumor-bearing mice in each group, *p <.05, ** *p* < .01. (F) Representative H&E and TUNEL staining of tumor tissues (Red: CD31, Blue: DAPI, Green: TUNEL; Scale bar: 100 μm).

The anti-tumor activities and biosafety of Taxol, PTX@TPGS-PBTE NPs, and RGD-PTX@TPGS-PBTE NPs were further evaluated in SCC-7 tumor-bearing mice (Figure 7A). The changes in the tumor volume and tumor weight indicated that Taxol had weak tumor inhibition ability with TIR of 49.24%, while PTX@TPGS-PBTE NPs and RGD-PTX@TPGS-PBTE NPs were more effective with the TIR of 72.10% and 84.90%, respectively (Figure 7B–D). Additionally, there were no obvious weight changes in any of the treatment groups (Figure 7E). This suggests that the NPs exerted little systemic toxicity. H&E staining and TUNEL staining assays were used to evaluate tumor-site apoptosis after treatment. As demonstrated in Figure 7F, tumors treated with RGD-PTX@TPGS-PBTE NPs showed extensive necrosis and severe karyopyknosis. Notably, the RGD-PTX@TPGS-PBTE NPs exhibited distinct green fluorescence, which reflects severe tissue apoptosis. These results indicated that RGD-PTX@TPGS-PBTE NPs had an excellent anti-tumor effect due to the active targeted delivery and ROS-responsibility properties, which resulted in efficient drug delivery into tumor cells.

**Figure 8.**
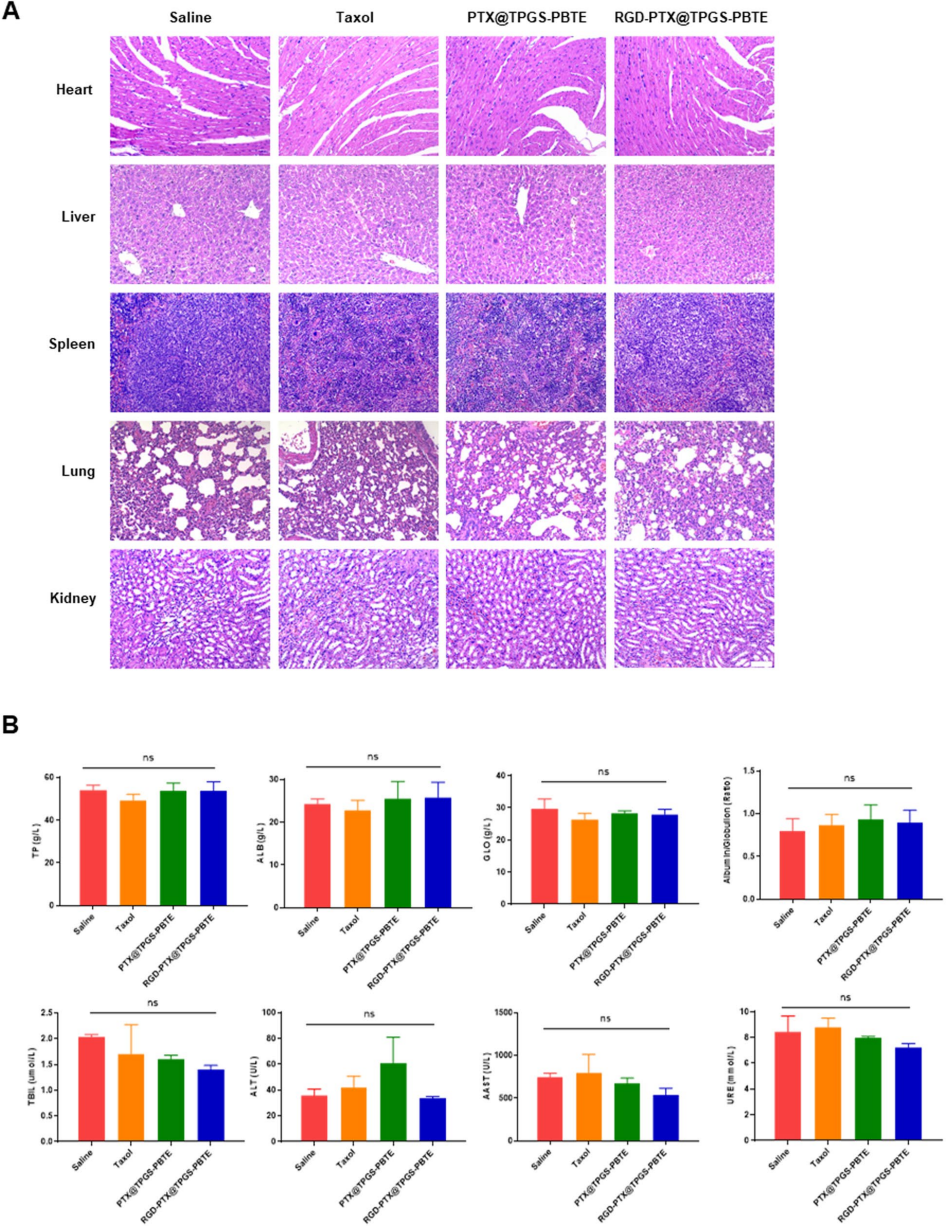
Safety evaluation. (A) Representative H&E staining of the major organs of SCC-7 tumor-bearing mice treated with various NPs. Scale bar: 100 μm. (B) Blood biochemical assessment.

The biosafety was further evaluated via H&E staining of the main organs and blood biochemical indexes. As shown in Figure 8A, the H&E staining of the major organs did not reveal any appreciable abnormalities in any of the groups, suggesting negligible toxicity of PTX@TPGS-PBTE NPs and RGD-PTX@TPGS-PBTE NPs to the major organs. The hematological parameters were all within the normal reference range (Figure 8B). This indicated that the PTX@TPGS-PBTE NPs and RGD-PTX@TPGS-PBTE NPs did not produce significant hepatic or renal dysfunction. Together, these results demonstrate that PTX@TPGS-PBTE NPs and RGD-PTX@TPGS-PBTE NPs had a good biosafety profile as a potential therapeutic agents.

## 4. Conclusion

In summary, an effective ROS-responsive drug delivery system based on PTX@TPGS-PBTE NPs was developed for PTX delivery. This system achieved accelerated drug release in the tumor microenvironment due to the relatively higher ROS concentration. In addition, cRGD-modified PTX@TPGS-PBTE NPs exhibited substantial accumulation in the tumor site, as compared with Taxol and PTX@TPGS-PBTE NPs due to a combination of active targeting mechanisms. Further, cRGD-modified PTX@TPGS-PBTE exhibited a superior anti-cancer effect and a notably enhanced tumor inhibition ratio. Together, these findings indicated that TPGS-PBTE NPs can serve as an efficient drug delivery system for the treatment of tumors in the future.

## Supplementary Material

Supplemental Material
