# Supplementary material for: Paclitaxel-loaded ROS-responsive nanoparticles for head and neck cancer therapy
Source: Drug Deliv. 2023 Mar 14;30(1):2189106. doi: 10.1080/10717544.2023.2189106 (PMC10026753; doi:10.1080/10717544.2023.2189106)

**Fig S1. The quantitative analysis of fluorescence intensity (n = 3). ** *p* < 0.01**

**
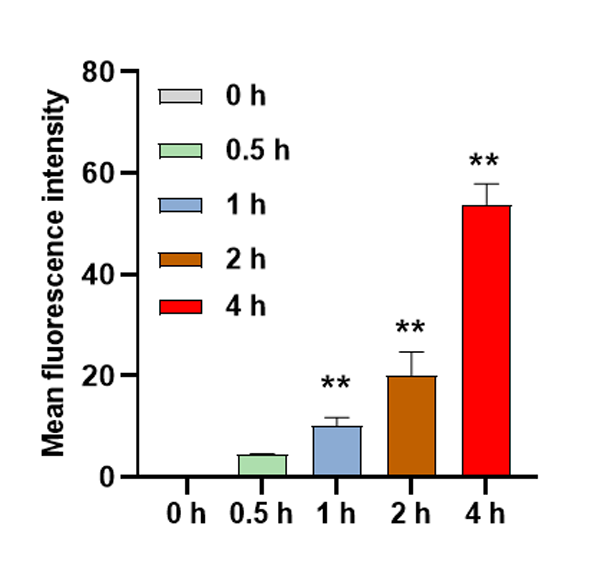
**

**Fig S2. The intracellular ROS in SCC-7 cells.**


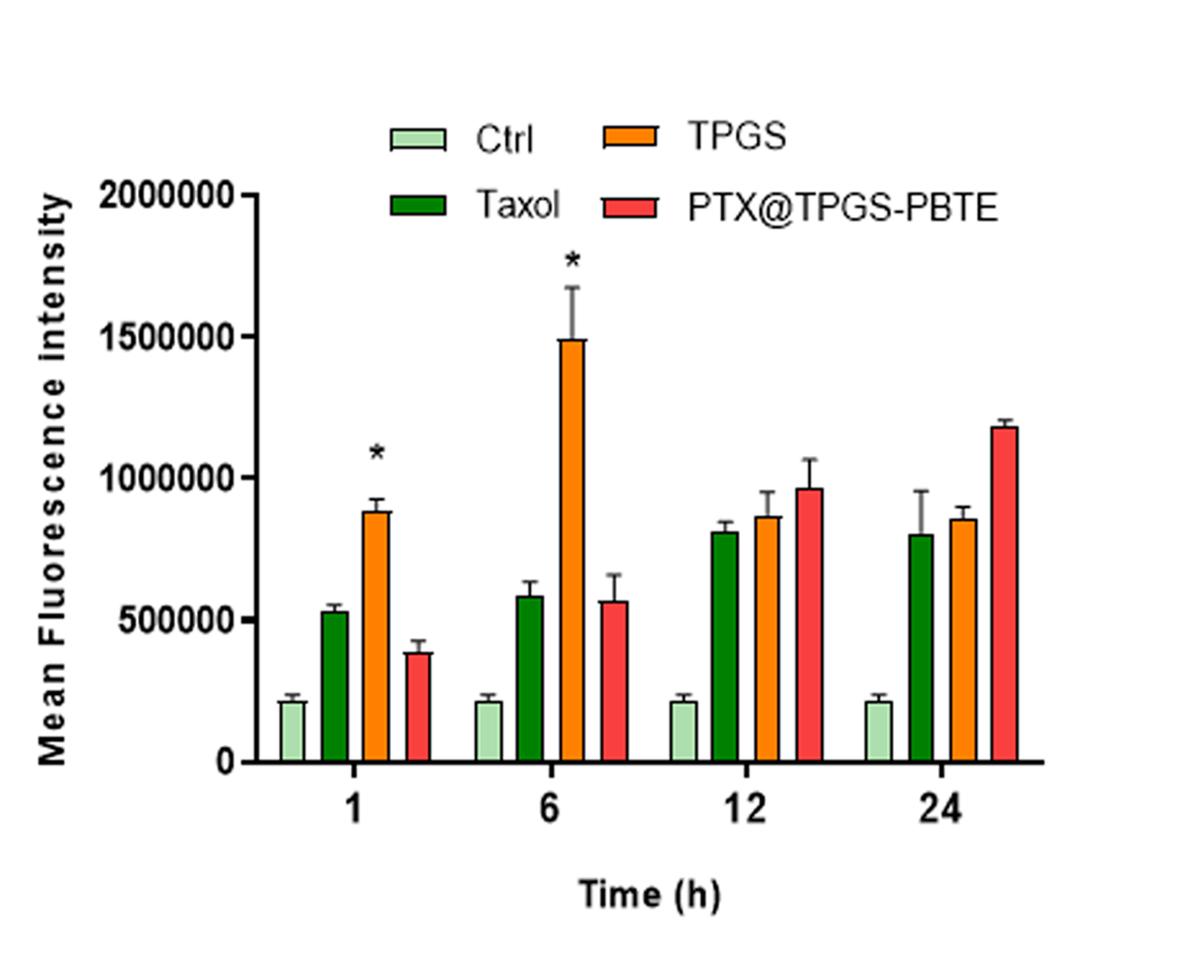

Supplement: Supplemental Material [file IDRD_A_2189106_SM5228.docx]
